# Supplementary figures and images for: A Transcriptomics Analysis of the Regulation of Lens Fiber Cell Differentiation in the Absence of FGFRs and PTEN
Source: Cells. 2024 Jul 19;13(14):1222. doi: 10.3390/cells13141222 (PMC11274593; doi:10.3390/cells13141222)

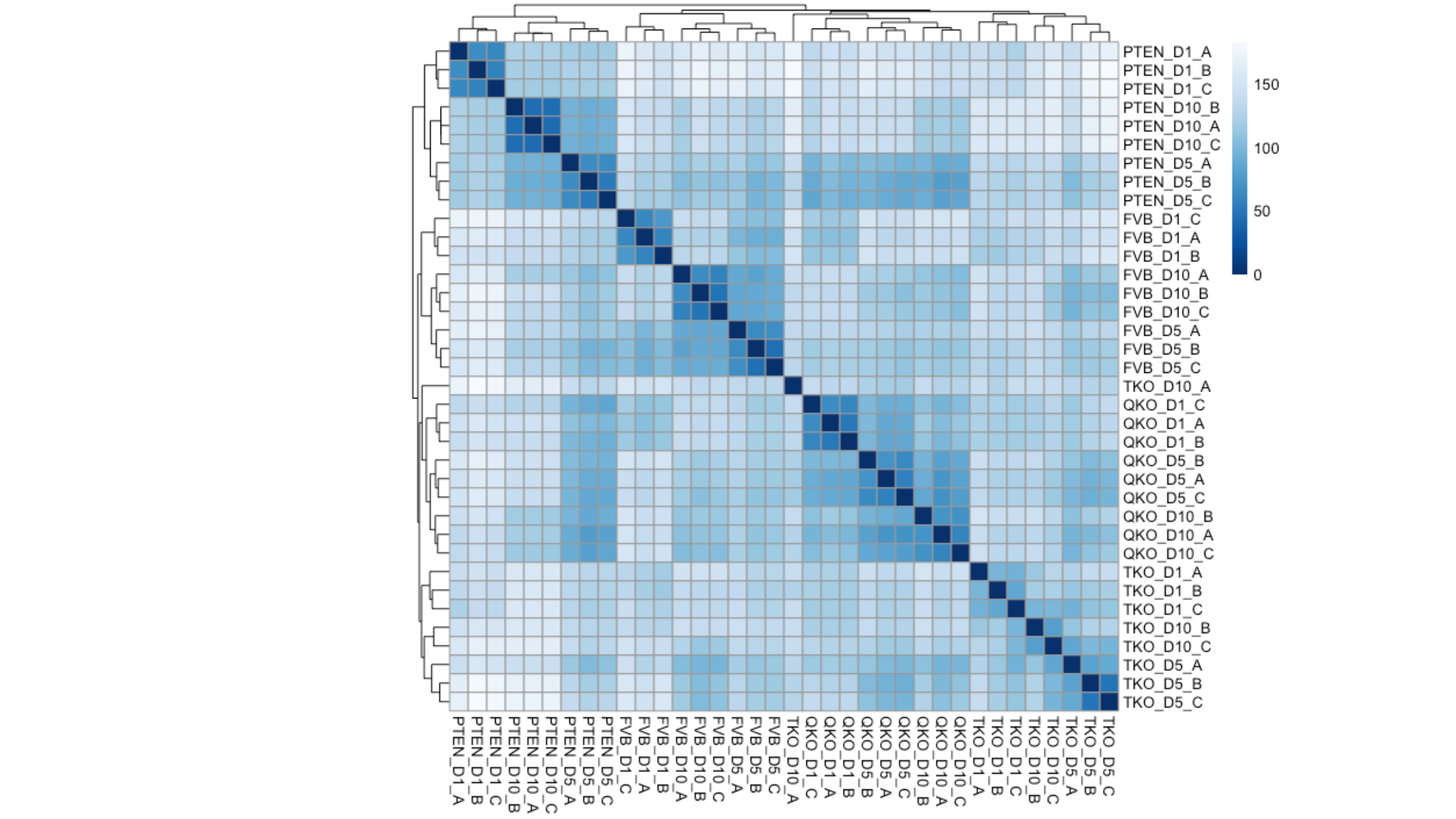

Supplement: Supplementary file 1 [file cells-13-01222-s001.zip › drive-download-20240627T212928Z-001/Figure S1.tiff]

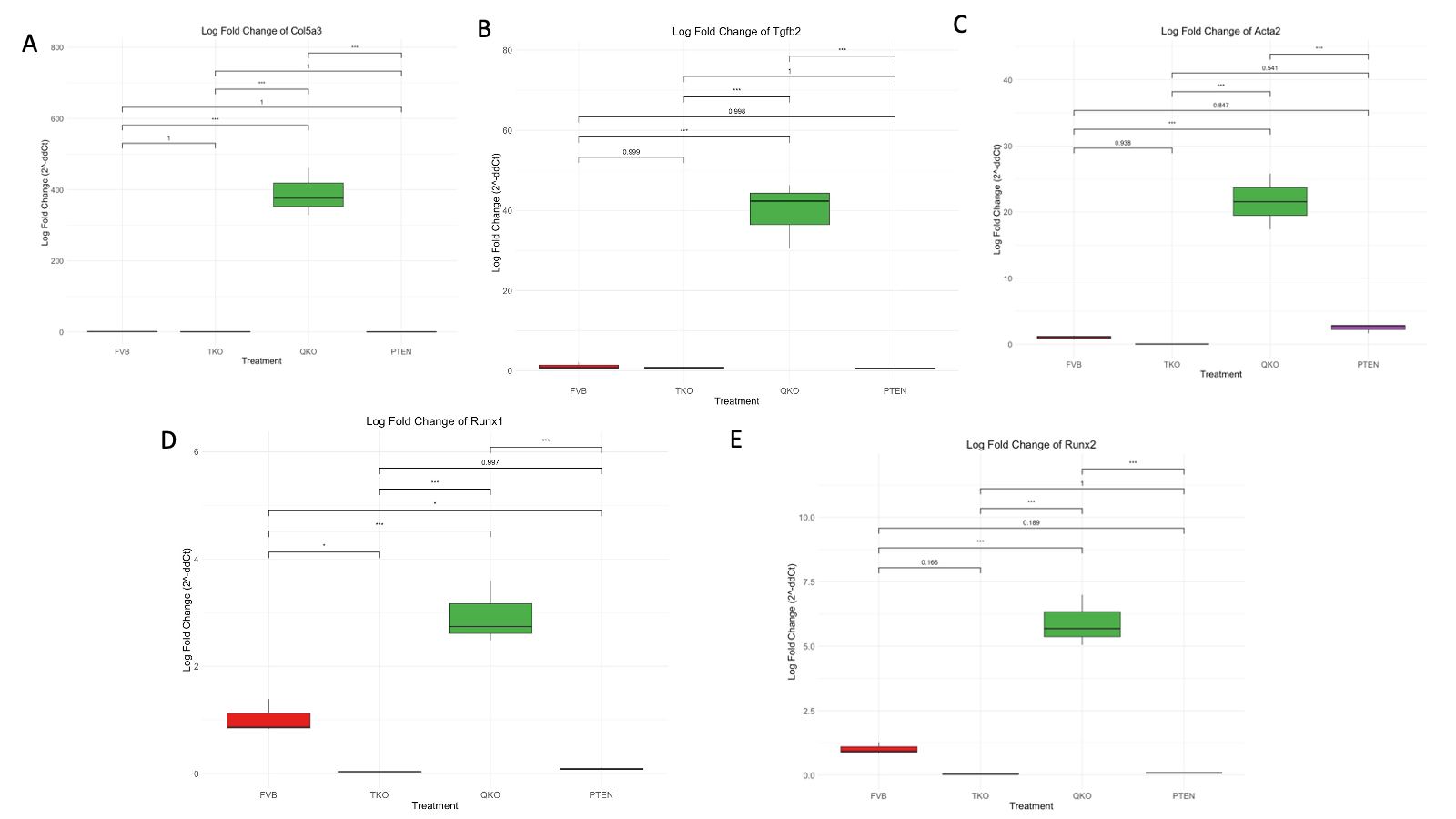

Supplement: Supplementary file 1 [file cells-13-01222-s001.zip › drive-download-20240627T212928Z-001/Figure S10.tiff]

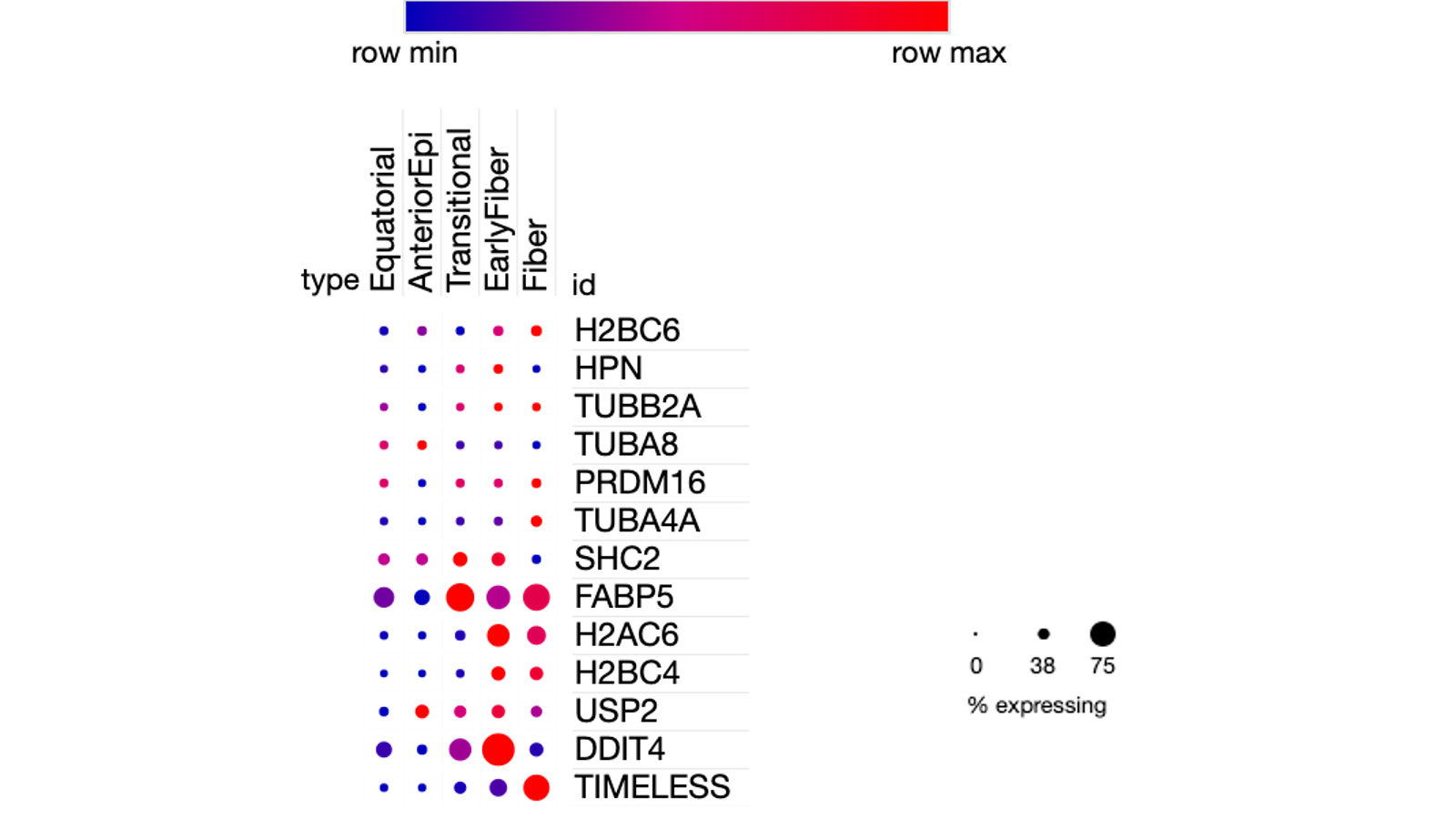

Supplement: Supplementary file 1 [file cells-13-01222-s001.zip › drive-download-20240627T212928Z-001/Figure S11.tiff]

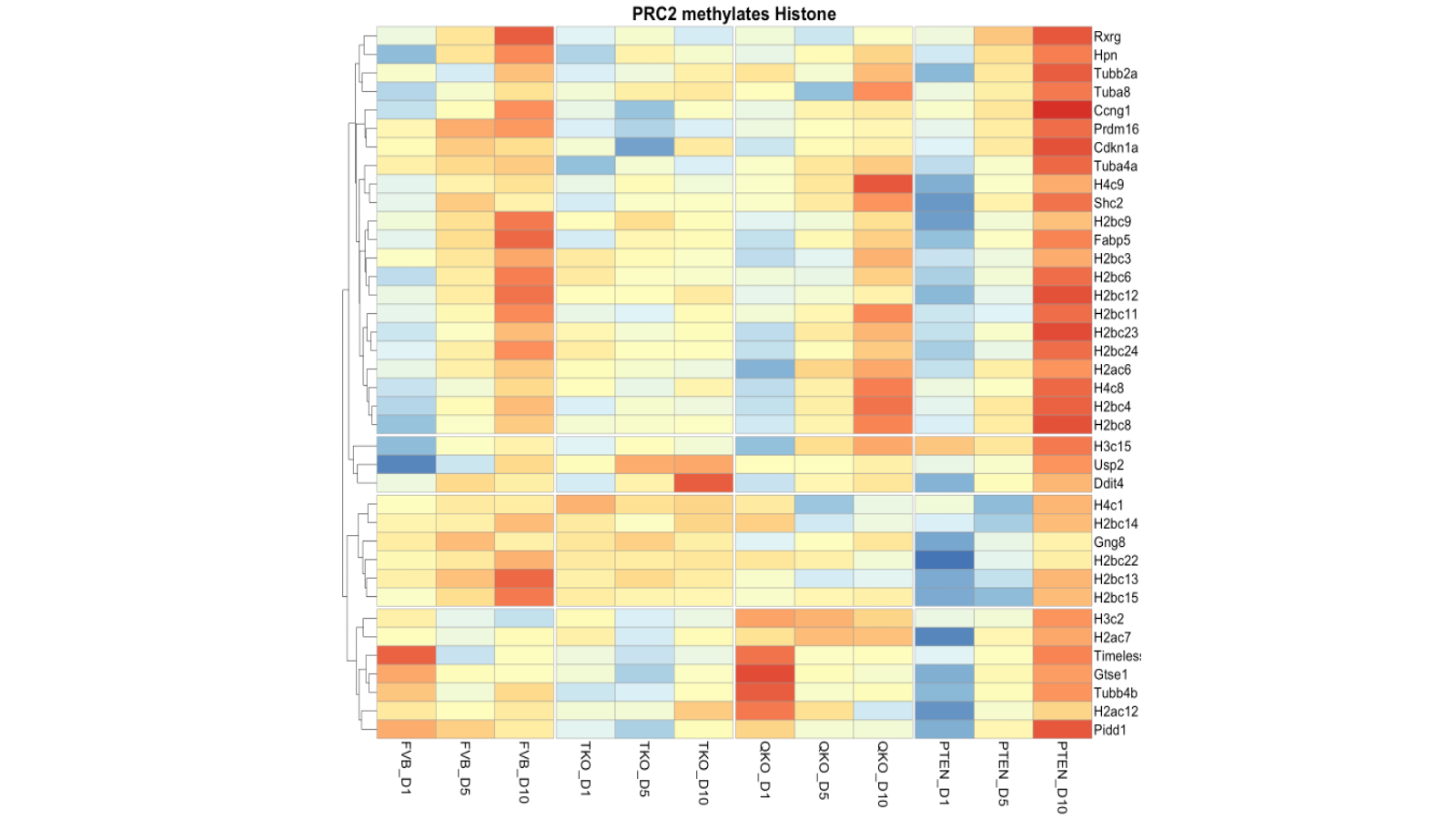

Supplement: Supplementary file 1 [file cells-13-01222-s001.zip › drive-download-20240627T212928Z-001/Figure S2.tiff]

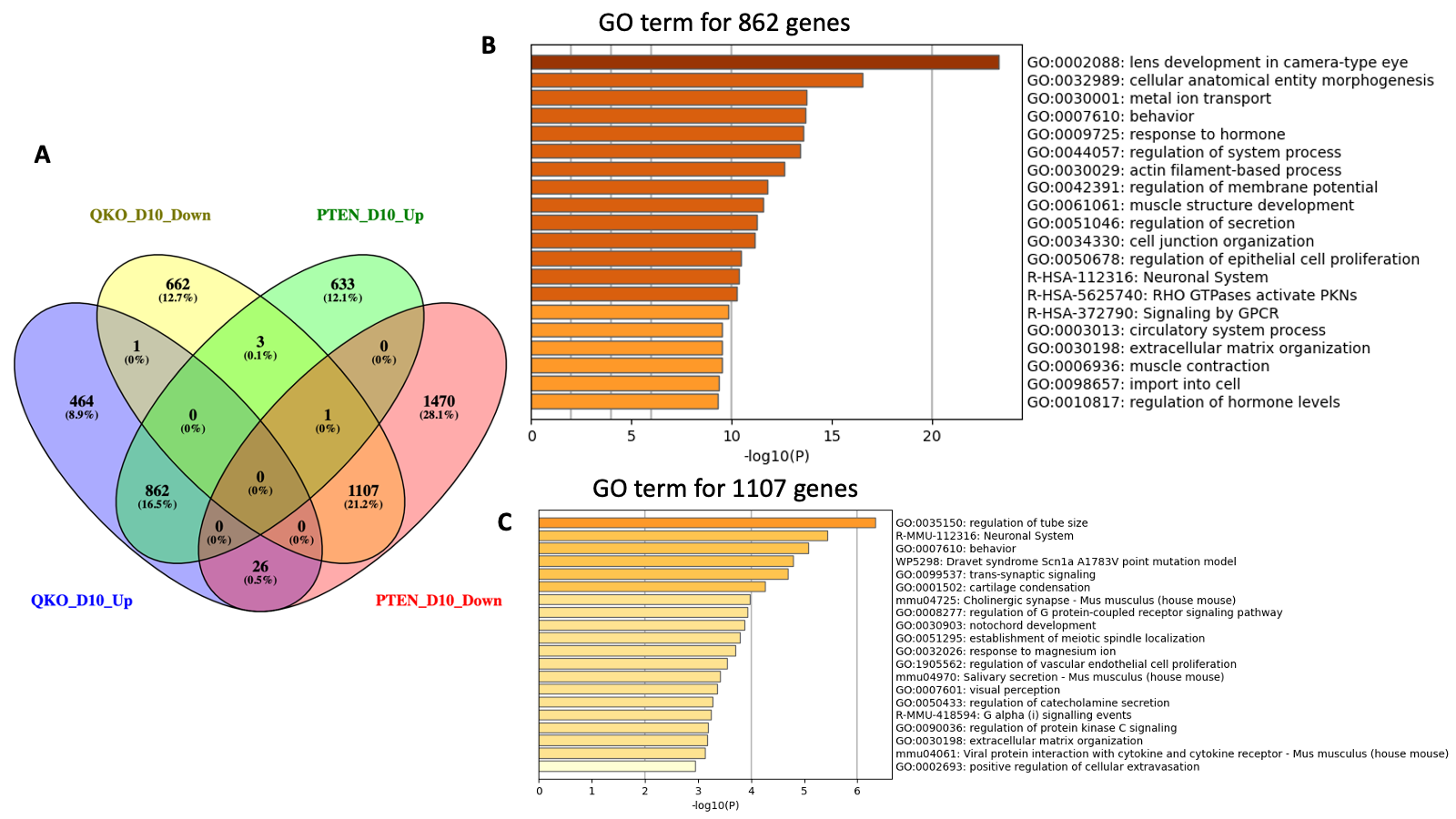

Supplement: Supplementary file 1 [file cells-13-01222-s001.zip › drive-download-20240627T212928Z-001/Figure S3.tiff]

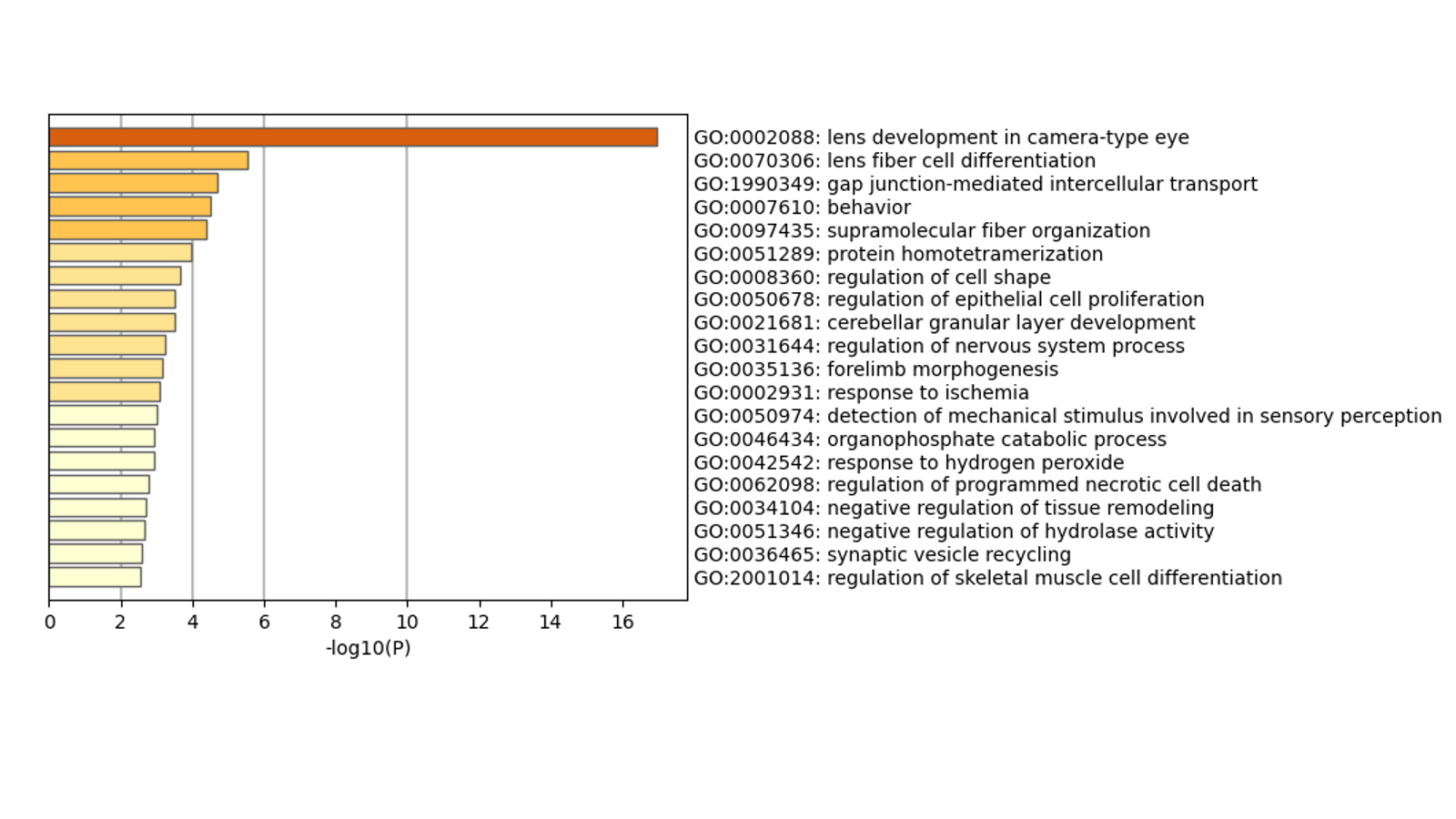

Supplement: Supplementary file 1 [file cells-13-01222-s001.zip › drive-download-20240627T212928Z-001/Figure S4.tiff]

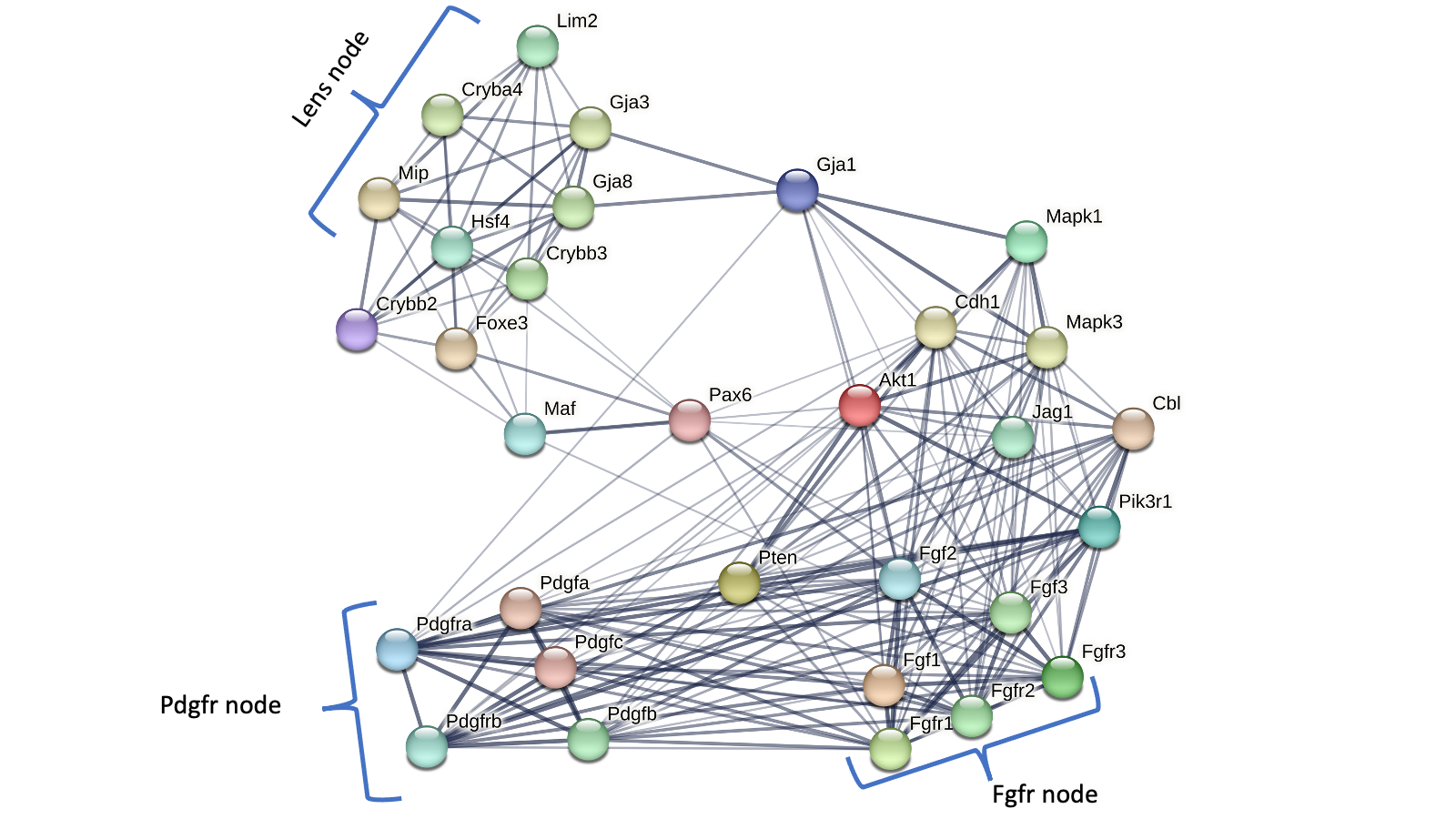

Supplement: Supplementary file 1 [file cells-13-01222-s001.zip › drive-download-20240627T212928Z-001/Figure S5.tiff]

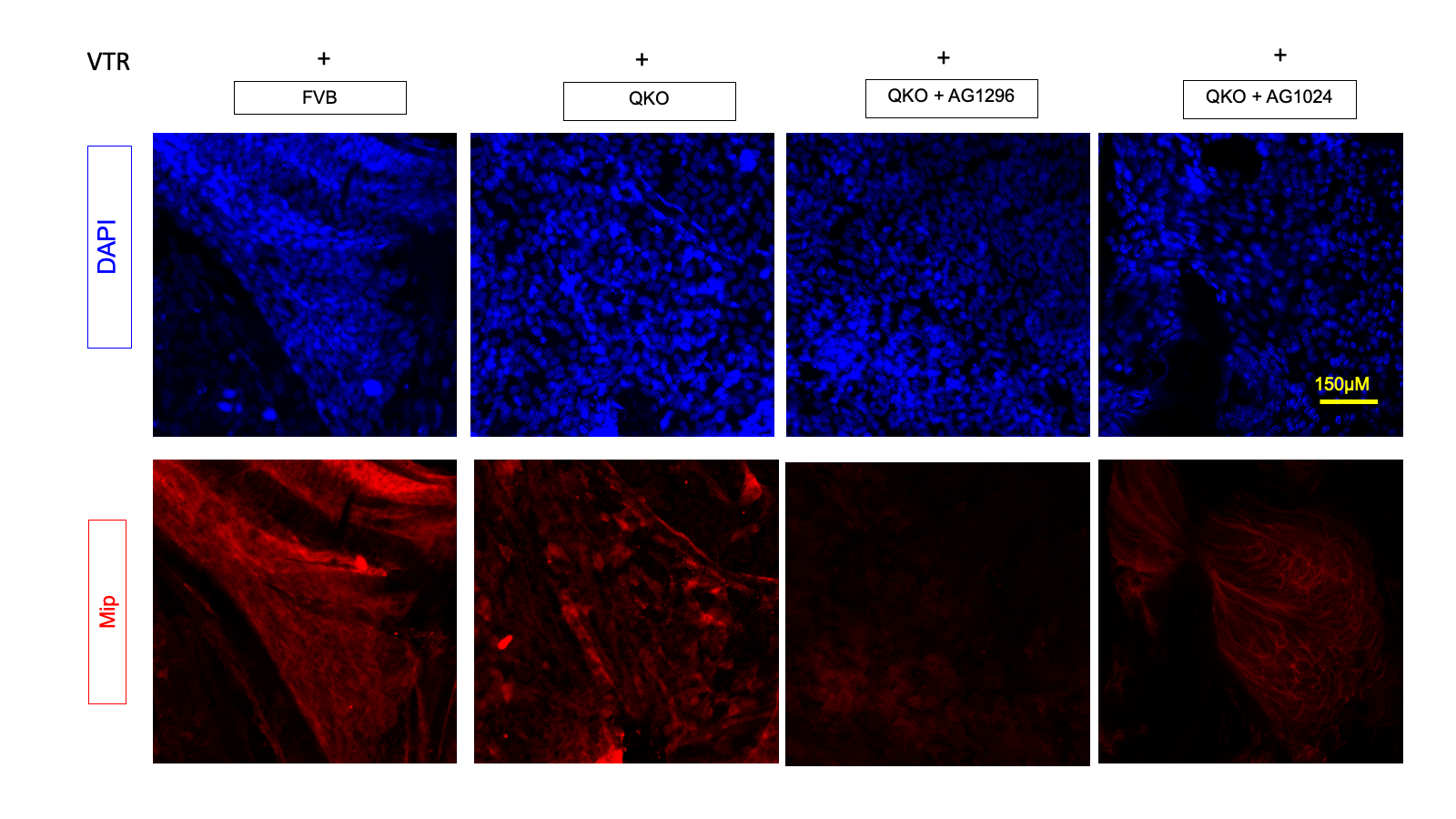

Supplement: Supplementary file 1 [file cells-13-01222-s001.zip › drive-download-20240627T212928Z-001/Figure S6.tiff]

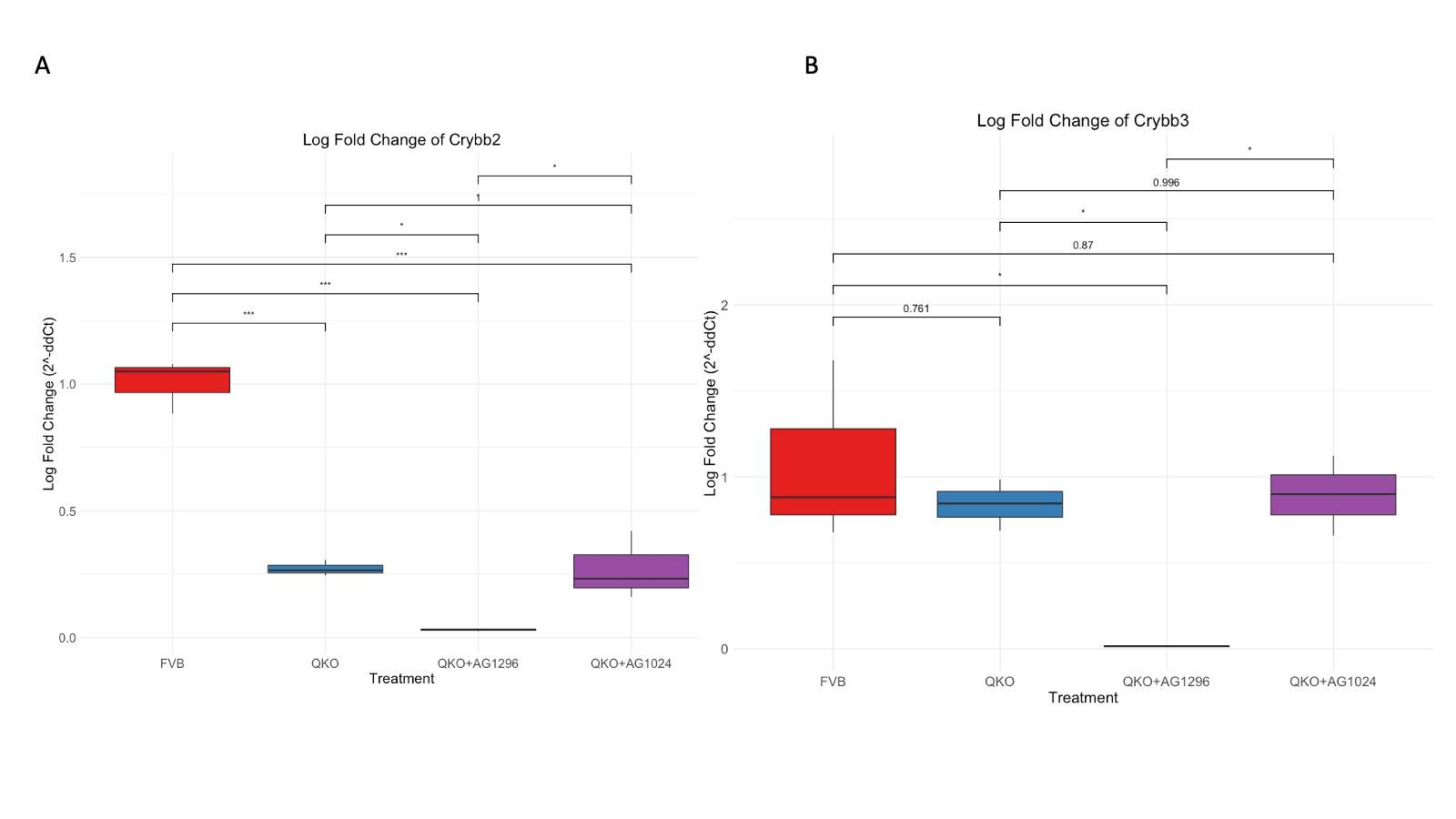

Supplement: Supplementary file 1 [file cells-13-01222-s001.zip › drive-download-20240627T212928Z-001/Figure S7.tiff]

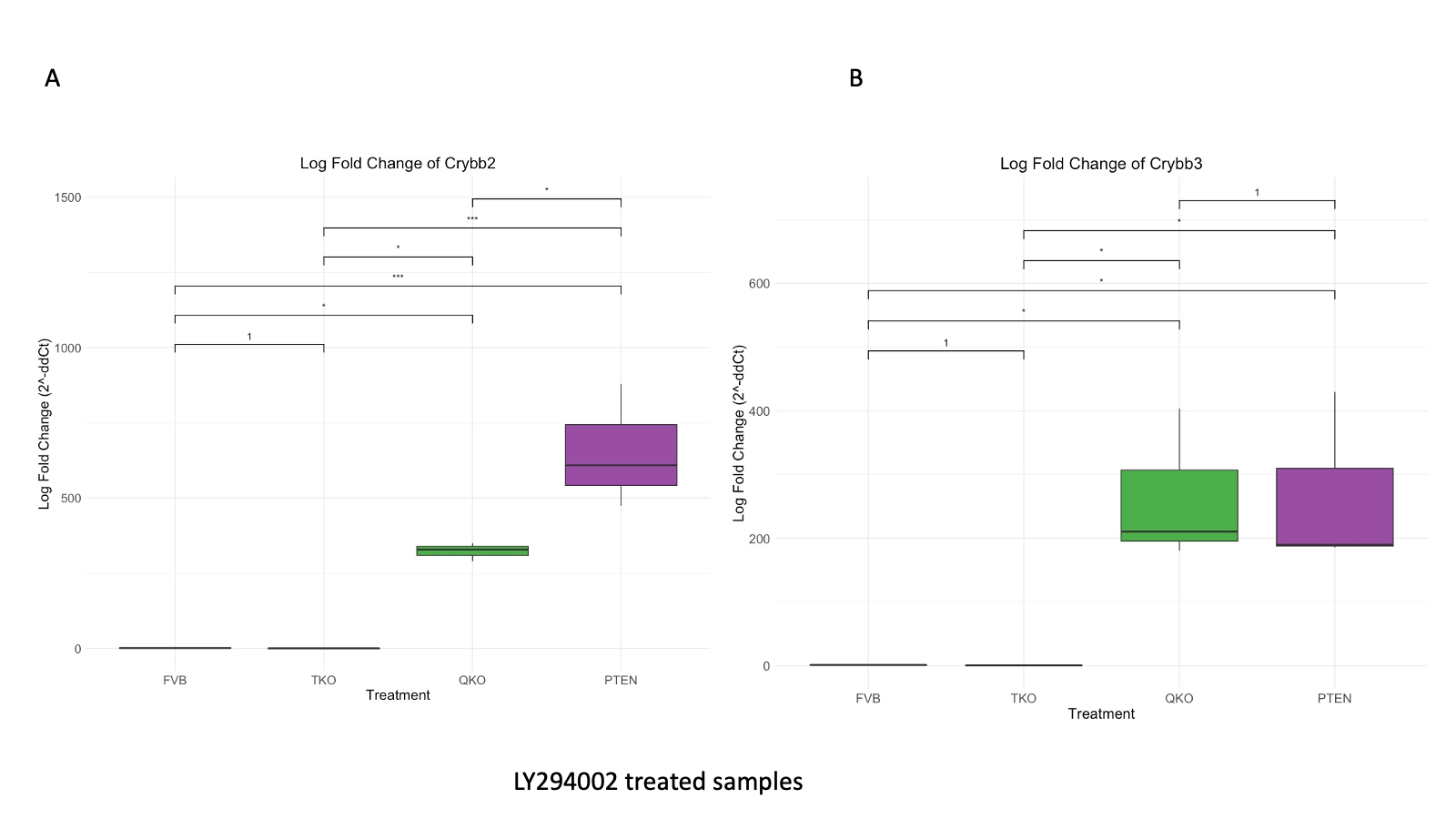

Supplement: Supplementary file 1 [file cells-13-01222-s001.zip › drive-download-20240627T212928Z-001/Figure S8.tiff]

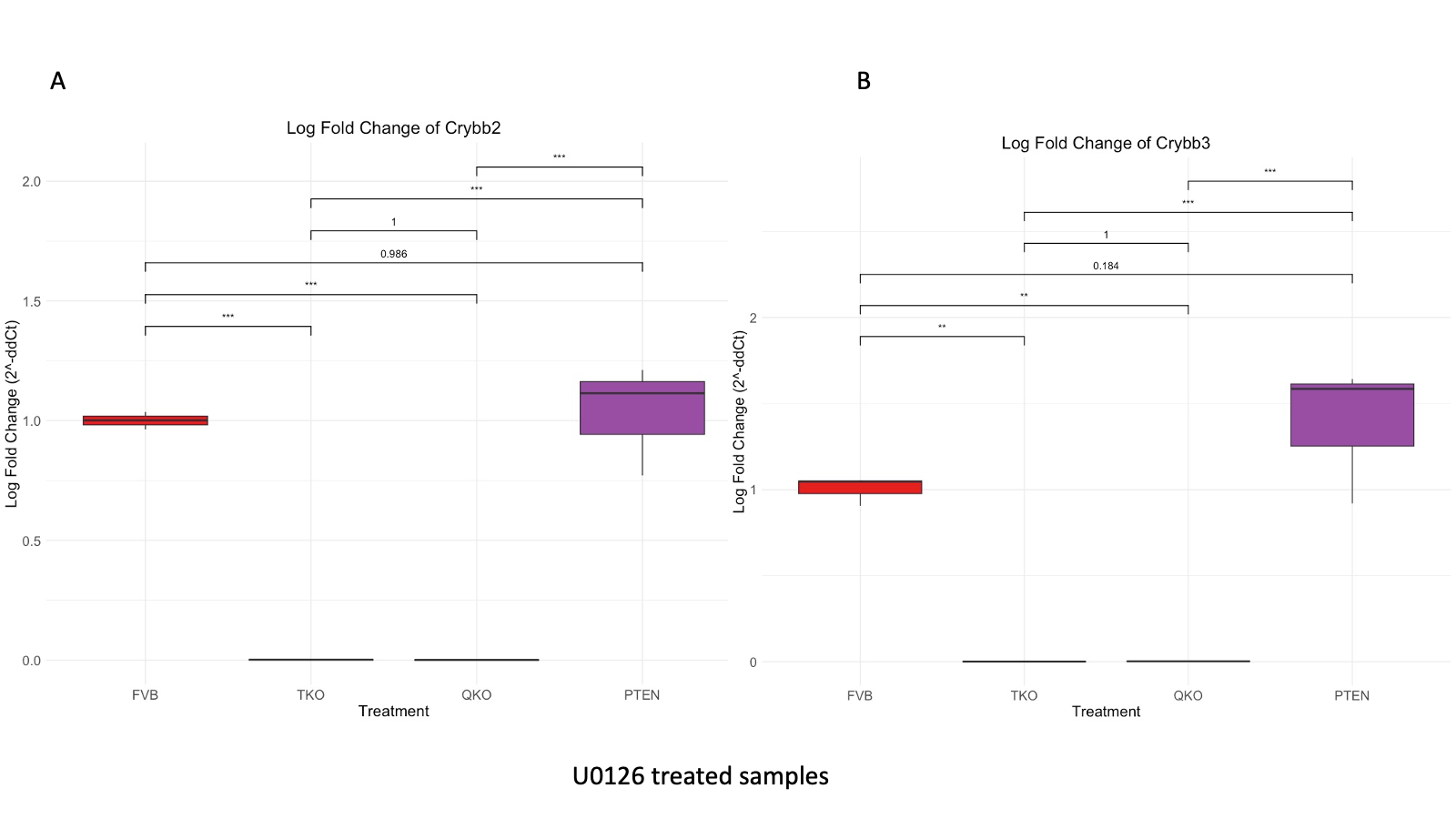

Supplement: Supplementary file 1 [file cells-13-01222-s001.zip › drive-download-20240627T212928Z-001/Figure S9.tiff]
